# Alcohol and Other Substance Screening in Bariatric Surgery Candidates: Utility of Self-Report and Toxicology Tests, Including Ethyl-Glucoronide

**DOI:** 10.1007/s11695-025-07774-z

**Published:** 2025-03-12

**Authors:** Silvia Cañizares, Laura Nuño, Pablo Barrio, Mireia Forner-Puntonet, Carolina Gavotti, Miquel Monràs, Patricia Gavín, Ricard Navinés, Lilliam Flores, Maite Barrios, Alba Andreu, Judit Molero, Amanda Jimenez, Josep Vidal, Anna Lligoña

**Affiliations:** 1 Section of Clinical Health Psychology, Department of Psychiatry and Psychology, Clinical Institute of Neurosciences (ICN), Hospital Clinic of Barcelona, C/Roselló 140, 08036 Barcelona, Spain; 2 Department of Clinical Psychology and Psychobiology, Universitat de Barcelona, Edificio Ponent Vall d’Hebron, 171 08035 Barcelona, Spain; 3 Addiction Unit, Clinical Institute of Neurosciences (ICN), Hospital Clinic of Barcelona, C/Villarroel, 170, 08036 Barcelona, Spain; 4 Sant Pau Mental Health Group, Department of Psychiatry, Hospital de La Santa Creu i Sant Pau, Institut de Recerca Sant Pau (IR Sant Pau), C/Sant Quintí, 89, 08025 Barcelona, Spain; 5 Department of Psychiatry and Psychology, Clinical Institute of Neurosciences (ICN, Hospital Clinic of Barcelona, C/Roselló 140, 08036 Barcelona, Spain; 6 Obesity Unit, Endocrinology and Nutrition Department, Hospital Clinic of Barcelona, C/Villarroel, 170 08036 Barcelona, Spain; 7 Centro de Investigación Biomédica en Red de Diabetes y Enfermedades Metabólicas Asociadas (CIBERDEM), Barcelona, Spain; 8 Department of Methodology of Sciences of the Behaviour, University of Barcelona, Edifici Ponent, Passeig de La Vall d’Hebron, 171, 08035 Barcelona, Spain

**Keywords:** Bariatric surgery, Obesity, Alcohol, Drugs, Ethyl-glucuronide, Risky drinking, AUDIT-C, ASSIST, Toxicology

## Abstract

**Abstract:**

Following bariatric surgery (BS) patients have an increased risk of alcohol misuse.

**Purpose:**

This 1-year cross-sectional study in potential BS candidates had several objectives: (a) assess the prevalence of risky drinking, alcohol use disorder (AUD), and other substance use/disorder; (b) compare the prevalence of these behaviors to that of the general Spanish population; (c) determine the proportion of patients with positive results in toxicology tests; and (d) study the predictive factors of risky drinking. Setting: tertiary university hospital.

**Materials and Methods:**

Alcohol and other substance use were evaluated with the AUDIT-C and ASSIST questionnaires. Urine tests analyzed several markers (ethyl-glucoronide [EtG] ≥ 500 ng/ml, amphetamine, benzodiazepine, cannabinoid, cocaine, and opioid). The Mini-International-Neuropsychiatric-Interview (5.0.0) was employed to assess psychiatric diagnoses.

**Results:**

Among 308 candidates for BS, 196 were accepted to participate (69% women; mean age 46.7 ± 10.9 years; mean body mass index 45.6 ± 5.9). AUDIT-C and ASSIST identified 7% and 5% of risky drinkers, respectively. Men were more frequently risky drinkers compared to women (18% vs. 2%) and compared to the general population (18% vs. 8%). Six percent of individuals had AUD, being men the most affected, and 2% met criteria for other substance disorder. Fifteen percent of the sample presented risky tobacco use. Cannabis was self-reported only by males (3%). EtG ≥ 500 ng/ml was present in 15% of the sample, being a risk factor for risky drinking together with the male sex.

**Conclusion:**

Identification of candidates at risk for risky drinking can help to prevent any alcohol misuse after BS. The combination of subjective and objective measures improves the validity of the assessment of substance use.

**Graphical Abstract:**

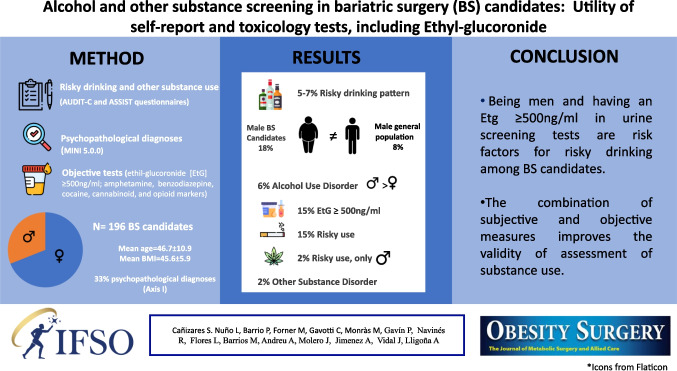

**Supplementary Information:**

The online version contains supplementary material available at 10.1007/s11695-025-07774-z.

## Introduction

Obesity has reached epidemic proportions in most developed societies. Bariatric surgery (BS) is the most effective method to lose and maintain long-term weight loss [1]. The relationship between BS and other causes of morbidity and mortality, such as alcohol use, has gained attention over the last years [2]. Preoperative psychological evaluation of BS candidates must include an assessment of the use of alcohol and other substances. Even in the absence of a formal alcohol use disorder (AUD), alcohol use is still relevant due to its negative influence on weight loss and nutritional status [3] and the increased risk of developing an AUD after BS in a subset of subjects [4,5]. Other substance use has received little attention in BS candidates [6,7].

Several studies have evaluated the use of alcohol in BS patients, with inconsistent findings, partly due to the heterogeneity of methodological aspects and the use of particular or ad hoc definitions of risky drinking and AUD [4,8–10].

Risky drinking is usually defined as the consumption of alcoholic beverages above the recommendations of national guidelines or alcohol risk screening tools [11]. This pattern generally involves the consumption of > 2 standard drinks per day and/or > 4 on a single occasion (binge drinking) and increases the likelihood of developing an AUD. According to the fifth edition of the Diagnostic and Statistical Manual of Mental Disorders (DSM-V) (American Psychiatric Association 2013), the diagnosis of AUD requires meeting several symptom criteria (impaired consumption control, social impairment, risky use, and tolerance and withdrawal syndrome), with a wide range of severity.

Alcohol biomarkers can objectively confirm alcohol intake increasing the validity of any alcohol assessment. In recent years, some direct ethanol metabolites, such as ethyl glucuronide (EtG) or phosphatidylethanol (Peth), have been used to reveal lighter patterns of alcohol consumption within a wider window of detection [12]. To our knowledge, only three studies using Peth have been conducted in BS patients [13–15].

## Purpose

The present study had four objectives: (1) assess the prevalence of risky drinking, AUD, and other substance use in BS candidates; (2) compare these prevalences with those of the general Spanish population; (3) determine the proportion of patients with high EtG values and those testing positive for amphetamines, benzodiazepines, cannabinoids, cocaine, and opioids in urine tests; and (4) determine the predictive factors of risky drinking.

## Materials and Methods

A cross-sectional study included participants evaluated for potential primary BS in the Obesity Unit of a tertiary hospital, from January to December 2017. After their first endocrinological visit, the patients were referred to a clinical psychologist who explained the study characteristics, and written informed consent was obtained before starting the psychological assessment visit and study evaluation, both being performed on the same day. The study was approved by the hospital Ethics Review Board (HCB/2016/0666).

The psychopathological diagnoses were made using the Spanish version of the Mini International Neuropsychiatric Interview (MINI) 5.0.0 which covers Axis I of the Diagnostic Statistical Manual IV [16].

In addition, current and past patterns of substance consumption were assessed with the following validated Spanish questionnaires:

- The AUDIT-C (Alcohol Use Disorders Identification Test Consumption), for screening risky alcohol consumption
- ASSIST (Alcohol, Smoking and Substance Involvement Screening Test), to detect substance use and related problems. We focused only on the moderate-high risk score of the last 3 months for each substance.

After completing the questionnaires, a urine sample was immediately collected from each participant, and thus, the individuals were unaware of the drug examination before the visit. Urine analysis included EtG and direct determination of amphetamines, benzodiazepines, cannabinoids, cocaine, and opioids. EtG was analyzed with a commercially available enzyme immunoassay (EIA) method based on a new monoclonal antibody DRI-EtG EIA (Thermo Fisher Scientific Diagnostics, Hemel Hempstead, UK) with a clinical cutoff ≥ 500 ng/ml, which is able to detect ethanol in urine for up to 72 h, depending on the amount ingested [12].

Data regarding psychopharmacological treatment were obtained from the electronic medical records of the participants.

### Statistical Analysis

The socio-demographic and clinical characteristics of the participants were analyzed according to sex with *t* and *χ*^2^ tests, and the Fisher’s exact test when required. The same analyses were performed between included patients and those who refused to participate in the study. The *Z*-test was used to compare single proportions of risky substance use obtained in our data to population estimates.

The association between risky drinking and several factors, selected in accordance to literature on alcohol use, was evaluated using logistic regression hierarchical analyses. Models were built with an enter method, with usual parameters, introducing predictors in the following order: EtG (< 500 or ≥ 500 ng/ml), sex, age, psychiatric diagnosis (none vs. at least one diagnosis but not AUD), and marital status (married vs. single, divorced or widowed). The significance level was set at 5%. IBM SPSS Statistics 20.0 was used for the statistical analyses.

## Results

### Sociodemographic and Clinical Characteristics of the Sample

Among the 308 patients referred, 196 accepted to participate and completed the assessment (69% women; mean age 46.7 years [range 18–66]; mean body mass index (BMI) 45.64 [range 32.79–70.37]). According to sex, no differences were found in age, educational level, perceived socioeconomic status, marital status, or BMI (Table 1). There were no statistical differences between patients who agreed and who did not agree to participate regarding sex or age, although participants exhibited a slightly greater BMI (supplementary material).

**Table 1:** Socio-demographic, clinical, and psychiatric features of the sample

	**Men** *n* = 61 (31%)	**Women** *n* = 135 (69%)	**Total** *n* = 196	**Sex differences**	
				***t*/*χ***^**2**^	***p*-value**
***Age*** (mean, SD)	47.08 ± 11.25	46.53 ± 10.91	46.7 ± 10.99	.327	.744
***Educational level***					
Primary	15 (24.6%)	33 (24.4%)	48 (24.5%)	.562	.819
Secondary	38 (62.3%)	89 (65.9%)	127 (64.8%)		
Superior	8 (13.1%)	13 (9.6%)	21 (10.7%)		
***Perceived socioeconomic satus***					
Low	30 (49.2%)	78 (57.8%)	108 (55.1%)	1.255	.281
Medium	31 (50.8%)	57 (42.2%)	88 (44.9%)		
***Marital status***					
Married	36 (59.9%)	82 (60.7%)	118 (60.2%)	1.401	.520
Single	18 (29.5%)	31 (23%)	49 (25%)		
Divorced/widowed	7 (11.5%)	22 (16.3%)	29 (14.8%)		
***Body mass index**** (mean, SD)*	45.15 ± 11.25	45.86 ± 6.16	45.64 ± 6.08	.762	.447
***Psyhchiatric diagnoses (MINI 5.0.0)***					
***Any disorder or disorders***	18 (30%)	45 (33.6%)	63 (32.5%)	.243	.740
***Affective disorders***					
Major depressive disorder	10 (16.7%)	17 (12.7%)	27 (13.9%)	.548	.780*
Dysthymia	4 (6.7%)	12 (9%)	16 (8.2%)	.287	.459
Suicidal ideation	6 (10%)	18 (13.4%)	24 (12.4%)	.451	.502
Manic episode	0 (0%)	1 (0.7%)	1 (0.5%)	.450	1*
Hypomanic episode	1 (1.7%)	1 (0.7%)	2 (1%)	.344	.524*
***Anxiety disorders***					
Generalized anxiety disorder	5 (8.3%)	16 (11.9%)	21 (10.8%)	.559	.455
Panic disorder	5 (8.3%)	15 (11.2%)	20 (10.3%)	.367	.545
Agoraphobia	2 (3.3%)	8 (6%)	10 (5.2%)	.589	.727*
Social phobia	0 (0%)	3 (2.2%)	3 (1.5%)	1.364	.554*
Obsessive–compulsive disorder	0 (0%)	1 (0.7%)	1 (0.5%)	.450	.1*
***Substance use disorders***					
Alcohol abuse	6 (10%)	1 (0.7%)	7 (3.6%)	10.204	.004*
Alcohol dependence	3 (5%)	0 (0%)	3 (1.5%)	6.805	.029*
Drug abuse	2 (3.3%)	0 (0%)	2 (1%)	4.513	.095*
Drug dependence	1 (1.7%)	0 (0%)	1 (0.5%)	2.245	.309*
***Psychotic disorders***					
Psychotic disorder	1 (1.7%)	0 (0%)	1 (0.5%)	2.245	.309*
Affective disorder with psychotic symptoms	0 (0%)	1 (0.7%)	1 (0.5%)	.450	1*
***Eating disorders***					
Bulimia nervosa	0 (0%)		4 (2.1%)	1.829	.313*

^*^Fisher’s exact test

^**^MINI (Mini International Neuropsychiatric Interview) 5.0.0

*SD*, standard deviation

Table 1 also shows the psychopathological diagnoses using the MINI. Among the study participants, 33% met the criteria for a current Axis I disorder at assessment, being major depressive disorder the most common (14%). Regarding AUD, 4% met the criteria for abuse and 2% for dependence, being men more likely to present an AUD. Finally, drug abuse and drug dependence diagnoses were met by 1% and 1%, respectively.

With respect to psychopharmacological treatment, 32% of patients had a current prescription for antidepressants, 28% for benzodiazepines, 17% opioids, and 5% for antipsychotics.

### Self-Reporting of Substance Use

AUDIT-C identified 14(7%) risky drinkers, with a higher proportion of men compared to women (18% vs. 2% *p* < 0.001). Similarly, the ASSIST detected 10 (5%) risky drinkers, with no difference in relation to sex (Table 2).

**Table 2:** AUDIT-C, ASSIST, and urine toxicology test results reported by sex

	**Men** *n* = 60	**Women** *n* = 134	**Total** *n* = 194	**Sex differences**		
				***χ***^**2**^	**(*p*-value)**	
***AUDIT-C***						
Hazardous drinking	11 (18.0%)	3 (2.2%)	14 (7.1%)	15.835		< .001*
***ASSIST medium–high risk***						
Tobacco	10 (16.4%)	19 (14.1%)	29 (14.8%)	0.179		.672
Alcohol	5 (8.2%)	5 (3.7%)	10 (5.1%)	1.752		.186
Cannabis	6 (9.8%)	0 (0%)	6 (3.1%)	13.698		.001*
Cocaine	0 (0%)	0 (0%)	0 (0%)			
Amphetamines	0 (0%)	0 (0%)	0 (0%)			
Inhalants	0 (0%)	0 (0%)	0 (0%)			
Benzodiazepines	2 (3.3%)	7 (5.2%)	9 (4.6%)	.349		.723*
Hallucinogens	1 (1.6%)	1 (0.7%)	2 (1.0%)	.336		.527*
Opioids	1 (1.6%)	0 (0%)	1 (0.5%)	2.224		.311*
Others	0 (0%)	0 (0%)	0 (0%)			
***Urine toxicology tests***						
EtG ≥ 500 ng/ml	13 (21.3%)	16 (11.9%)	29 (14.8%)	2.982		.084
Benzodiazepines	4 (6.6%)	9 (6.7%)	13 (6.6%)	.001		1*
Amphetamines	0 (0%)	0 (0%)	0 (0%)			
Cannabis	6 (9.8%)	1 (0.7%)	7 (3.6%)	10.092		.004*
Cocaine	2 (3.3%)	1 (0.7%)	3 (1.5%)	1.796		.229*
Opioids	1 (1.6%)	1 (0.7%)	2 (1%)	.336		.527*

^*^Fisher’s exact test

*AUDIT-C*, Alcohol Use Disorders Identification Test Consumption; *ASSIST*, Alcohol Smoking and Substance Involvement Screening Test

Post hoc analyses showed deficient, albeit statistically significant, concordances between risky drinking according to self-reporting results and the EtG (AUDIT-C kappa 0.305, *p* < 0.001; ASSIST kappa 0.140, *p* = 0.021, respectively).

Tobacco was the drug most commonly used at a risky level in 15% of the sample, with no differences according to sex. Regarding other recreational substances apart from alcohol, cannabis was the most frequently self-reported (3%) only by men (*p* = 0.001). The use of non-prescribed benzodiazepines was recognized by 5% of the participants, with no differences according to sex, and neither were there differences in the use of hallucinogens or opioids. No individual declared cocaine, amphetamines, or inhalant use.

### Comparisons of Prevalence with the General Population

Men in our sample showed a greater prevalence of risky drinking than men in the general population according to AUDIT-C test (*z* = 3.2, *p* 0.0012) [17].

Risky tobacco and cannabis use was more prevalent in the general population than in our sample (*z* = 5.9, *p* < 0.0001; *z* = 4.2, *p* < 0.0001 respectively), while the use of hallucinogens was slightly higher in BS candidates (*z* = 3.4, *p* = 0.0007) [17]. There were no differences in the use of non-prescribed benzodiazepines, cocaine, amphetamines, or inhalants.

### Urine Screening Test Results

EtG values were ≥ 500 ng/ml in 29(15%) individuals with no differences according to sex (Table 2).

The cannabis marker was positive in 4% of the sample, with a significantly higher frequency in males compared to females (10% vs. 1%, *p* = 0.004). Other positive results in the overall urine screening sample were for benzodiazepines (7%), cocaine (2%), and opioids (1%) (Table 2).

### Predictors of Risky Drinking

The logistic regression model showed EtG (≥ 500 ng/ml) and sex (men) to be significant predictors of risky drinking according to AUDIT-C, with an odds ratio (OR) = 8.367 (95% confidence interval [CI] 2.042–34.274, *p* = 0.003) and an OR = 10.392 (95% CI 2.412–44.782, *p* = 0.002), respectively. In addition, marital status was found to be a protective factor (being single, divorced, or widowed) OR = 0.194 (95% CI 0.047–0.794) (*p* = 0.023). Neither age nor psychiatric diagnoses were significant. The area under the curve (AUC) was 0.896.

The only significant predictor of risky drinking according to ASSIST was EtG (≥ 500 ng/ml.), with an OR = 5.017 (95% CI 1.23–20.458) (*p* = 0.025) and AUC 0.713.

## Discussion

To our knowledge, this is the first study conducted on BS candidates that simultaneously describes the self-reporting of alcohol and substance use with objective urine toxicological tests. The overall prevalence of risky drinking in BS candidates was quite similar to that of the general population (7% vs. 5%, respectively) according to the AUDIT-C questionnaire. However, two additional relevant conclusions were derived from this study. First, we found a much higher proportion of male BS candidates with a risky drinking pattern compared to women BS candidates (18% vs. 2%). Second, male BS candidates showed a more than two-fold greater prevalence of risky drinking than men in the general Spanish population (18% vs. 7.6%, respectively)^(17)^.

In contrast, no differences according to sex were found with the ASSIST questionnaire, with a general prevalence of risky drinking of 5%. Although the AUDIT and ASSIST are screening tests, there are some differences in relation to item content and assessment time period that may account for these disparities.

Previous studies on risky drinking in BS candidates have yielded conflicting results. On one hand, the Longitudinal Assessment of Bariatric Surgery-2 [10] and Assessment of Bariatric Surgery studies [9] described a higher prevalence of risky drinking among BS candidates (19.6% and 16.6%, respectively), although data by sex were not provided. Nevertheless, these higher prevalences are likely overestimated due to methodological issues, since risky drinking was classified not only by the AUDIT score but also when the participant gave any positive response to additional or modified items suggestive of binge drinking, alcohol abuse, or dependency. Therefore, our data are not completely comparable to the results of these studies. On the other hand, the Swedish Obese Subject study [18] found no differences in risky drinking between BS candidates and matched controls. Nonetheless, this study had an important bias since participants who reported alcohol problems or with consumption ≥ 2.5 standard drinks were excluded. In summary, differences in assessment tools or criteria may account for the discrepant results found. It should be taken into account that BS candidates tend to underreport psychopathology or substance use in the pre-surgical assessment [19]. Indeed, most risky drinker individuals tend to underestimate their consumption and risk [20].

With regard to the alcohol biomarker, 15% of our patients presented EtG values ≥ 500 ng/ml, demonstrating that alcohol had been consumed within the previous 72 h. Surprisingly, alcohol biomarkers have seldom been used in BS candidates. Two studies using Peth, with a determination ≥ 0.05 µmols/L, reported similar proportions (12–18%) [13,14] as was also described in a retrospective study [15] in which 15.3% of individuals showed values of heavy alcohol use (≥ 200 ng/mL). Although an EtG ≥ 500 ng/ml is not diagnostic per se, in our study, this value increased the probability of being a risky drinker by 5 to eightfold. However, as expected, EtG and alcohol questionnaires were not concordant, similar to the previous studies with Peth, since different aspects were assessed with subjective and objective methods.

Nonetheless, the most significant predictive factor of risky drinking was sex in our study. Sex has previously been identified as an important risk factor for alcohol misuse [21]. Males seeking BS had a tenfold greater risk of risky drinking than females. Unexpectedly, being single, divorced, or widowed was a protective factor in our analysis, although the magnitude of its effect makes it negligible and the relationship between alcohol consumption and social support is complex [22]. Age and having any psychiatric diagnosis (apart from AUD) were not significant predictors of risky drinking in our study, in contrast to what some studies after BS have suggested [10].

The overall prevalence of AUD in the present study was 6%, quite similar to that obtained in other preoperative studies [10] using procedures not as robust as our structured criteria.

Assessment of alcohol use is relevant since an increased risk of developing any alcohol problem has been described, especially 2 years after BS. The risk is somehow greater in patients who had undergone Roux-en-Y-gastric bypass (RYGB)^16^, but also, it has been reported also in patients who had undergone sleeve gastrectomy [10,23,24], even in patients without a previous history of unhealthy alcohol use [25]. Anatomical and metabolic changes secondary to BS can modify the pharmacokinetics of alcohol, producing higher and longer blood concentrations for equivalent intakes prior to surgery, and thus with greater alcohol exposure in the brain [26]. There may also be some shared underlying physiological features between addiction and obesity in brain reward circuitry or its disruptions [27,28] and common genetic influences in both disorders [2] as well as a switch in coping strategies from eating to alcohol intake [29]. The Labs-2 study [21] reported a significant increase in regular drinking and in the prevalence of AUD after BS (especially following RYGB, increasing from 7% presurgery to 16% 7 years post-surgery), as well as a greater risk of illicit drug use.

All these findings have important clinical implications. BS patients, mainly males but also females, must be advised that alcohol use can increase or generate more negative health consequences after BS, even with amounts not considered harmful. This warning should be given to all patients, with or without any preoperative alcohol use, and should be maintained in the long term, since some studies [21] show significant figures up to 7 years after BS. This might be due to a certain relaxation of the patients over time with respect to their care. Therefore, regular monitoring is warranted, since besides the heightened risk of any type of harmful alcohol intake including AUD, alcohol use could potentially lead to poorer long-term BS outcomes, such as recurrent weight gain due to increased caloric intake from alcohol, malnutrition because of the possible compromise of adherence to nutritional guidelines and supplementation, higher rates of alcohol-related health complications including the risk of alcohol-associated liver disease [23] and mental health issues. Moreover, family, social, work, and economic consequences may also arise. The multidisciplinary team should be aware of any sign suggestive of alcohol-related difficulties to work together with the patient to adequately address the problem.

Apart from alcohol, tobacco was the drug most frequently self-reported at a risky level in 15% of the sample, similar to other studies (10 to 19%)^4,30^.

Regarding other recreational substances, the global prevalence of risky use was 9%, being somewhat higher than the 4.5% reported by previous research [10,30]. Cannabis use was objectively verified in 4% of the males in our sample. Urine tests detected slightly higher figures than self-assessment for the use of benzodiazepines without prescription, opioids for non-medical use, and cocaine, confirming the under-reporting in BS candidates. Since few studies in BS patients have focused on other substances beyond alcohol and tobacco, comparison with our results is difficult. A recent study performed toxicology screening [31] and found similar proportions for benzodiazepines (5.6 vs. 6.6% in our study) and a slightly higher proportion for amphetamines (1.2 vs. 0%). Nonetheless, our study detected somewhat higher positive results for cocaine (1.5 vs. 0%) and cannabinoid markers (3.6 vs. 1.8%). Finally, a very small proportion of participants met the criteria for drug dependence or abuse (1% and 1%, respectively).

Reliability and validity-related aspects are among the strengths of our study: a semi-structured psychiatric interview characterized the sample, the use of validated questionnaires and urine toxicology screening, and participants were unaware of the performed drug examination before the study assessment visit. This study also has some limitations, such as differences in the temporal dimension of the questionnaires and the fact that an EtG ≥ 500 ng/ml does not necessarily indicate a harmful or risky pattern of drinking. The voluntary nature of the study might have implied some selection bias in the sample, although participants and non-participants were equivalent in age and sex. However, we cannot say with certainty that patients who did not volunteer were not at increased risk for alcohol and other substance use.

## Conclusions

In summary, our study demonstrated that male BS candidates present a higher prevalence of risky drinking than female candidates and men in the general population. In addition, the presence of AUD was greater in male BS candidates compared to the female counterparts. Moreover, being male and having an EtG ≥ 500 ng/ml were risk factors for risky drinking. Urine toxicology tests increase the validity of substance evaluation. The use of other drugs, except tobacco, was much less prevalent. Monitoring individuals at risk to prevent further complications after BS is warranted. Future longitudinal studies are necessary to examine the use of substances after BS with reliable methods.

## Supplementary Information

Below is the link to the electronic supplementary material.

Supplementary file1 (DOCX 22 KB)

## Data Availability

The datasets generated during and/or analyzed during the current study are available from the corresponding author on reasonable request.
