# Supplementary material for: Alcohol and Other Substance Screening in Bariatric Surgery Candidates: Utility of Self-Report and Toxicology Tests, Including Ethyl-Glucoronide
Source: Obes Surg. 2025 Mar 12;35(4):1242–50. doi: 10.1007/s11695-025-07774-z (PMC11976782; doi:10.1007/s11695-025-07774-z)
Supplement: Supplementary file 1 — Supplementary file1 (DOCX 22 KB) [file 11695_2025_7774_MOESM1_ESM.docx]

**Supplementary material**

**Table 1.**  Socio-demographic characteristics of individuals who participated and who did not agree to participate in the study.

|  | **Patients Included**  (N=196) | **Patients**  **Not-included**  (N=112) | **Differences** | |
| --- | --- | --- | --- | --- |
|  |  |  | **t / χ^2^** | **P-Value** |
| Age (yrs.)  (mean, SD, range) | 46.7±10.99  (18-66) | 46.53±10.91  (21-66) | -.150 | .881 |
| *Sex (frequencies)* | | | | |
| Male | 61(31%) | 32(29%) | .268 | .605 |
| Female | 135(69%) | 80(71%) |  |  |
| Body Mass Index  (mean, SD, range) | 45.64±6.08  (32.79-70.37) | 44.1 ±5.15  (34.14-62.67) | 2.165 | .031 |
